# Solid γ-Cyclodextrin Inclusion Compound with Gingerols, a Multi-Component Guest: Preparation, Properties and Application in Yogurt

**DOI:** 10.3390/biom10020344

**Published:** 2020-02-22

**Authors:** Joana M. Pais, Bruna Pereira, Filipe A. Almeida Paz, Susana M. Cardoso, Susana S. Braga

**Affiliations:** 1 QOPNA & LAQV/REQUIMTE, Chemistry Department, University of Aveiro, 3810-193 Aveiro, Portugal; joanampais@ua.pt (J.M.P.); bruna.filipa@ua.pt (B.P.); susanacardoso@ua.pt (S.M.C.); 2 CICECO – Aveiro Institute of Materials, Chemistry Department, University of Aveiro, 3810-193 Aveiro, Portugal; filipe.paz@ua.pt

**Keywords:** fresh ginger rhizome, molecular encapsulation, antioxidant, food fortification, nutraceutics

## Abstract

Gingerols from the rhizome of fresh ginger (*Zingiber officinale*) were obtained by a simple extraction, followed by purification. The gingerols extract was composed of 6-gingerol (54%), 8-gingerol (20%), and 10-gingerol (26%). It was included into γ-cyclodextrin by classic co-dissolution procedures. Solid-state characterisation of γ-cyclodextrin·gingerols shows that this inclusion compound features 1:1 host-to-guest stoichiometry and that it is a microcrystalline powder with a crystalline cell that belongs to the tetragonal space group 42_1_2, having the host molecules stacked in infinite channels where the gingerols are accommodated. *In chimico* studies with ABTS^•+^ scavenging, NO^•^ scavenging, β-carotene peroxidation, and 5-LOX inhibition show that γ-cyclodextrin is a suitable carrier for gingerols, because it does not alter their reactivity towards these substances. Yogurt was tested as a matrix for the incorporation of gingerols and γ-cyclodextrin·gingerols into foodstuff. The colour of the fortified yogurt suffered little alterations. In the case of yogurt with the inclusion compound, γ-cyclodextrin·gingerols, as fortificant, these alterations were not perceptible to the naked eye. Moreover, yogurt with γ-cyclodextrin·gingerols showed a good antioxidant activity, thus being suitable for use in nutraceutical applications.

## 1. Introduction

Ginger, or *Zingiber officinale*, is the rhizome of a perennial herb that is widely used in Asia in Traditional Medicine. Across the globe, it is recognized for its food applications, being used as a spice and as an ingredient in beverages, such as ginger infusion and ginger ale. In the United States, ginger has the GRAS status (‘generally recognized as safe’) and it can be consumed without any dose restrictions [1]. Data from 2017 indicated a global production of ginger of 3,038,120 tons, with India, China, and Nigeria being the three top producers [2]. Ginger is traditionally associated with the amelioration of nausea and indigestion, while, in ayurvedic medicine, it is considered to be a universal medicine. However, clinical studies on humans show that ginger is not a panacea and its proven effects are restricted to the prevention of motion sickness, the alleviation of dysmenorrhea-associated pain, and the modulation of inflammatory conditions, such as strain-induced muscle pain and early stages of ostheoarthritism [3] Further activities of ginger are currently under investigation and warrant further data to support claimed benefits. A relevant example is the gastroprotective action, as demonstrated by various studies in rodents (mice, rats, and Mongolian gerbils). These studies show that ginger avoids ulceration that is induced by stimuli, such as stress [4] or *Helicobacter pilori* infection [5,6], and chemical agents, like alcohol [4,7,8] or NSAIDS (non-steroid anti-inflammatory drugs) [4,9]. However no data on humans are available regarding ginger’s gastroprotective action. Further investigation is also needed on anti-diabetic action, as reported by few clinical trials, but still lacking longitudional studies, and on the possible activities against inflammatory bowel disease and fatty liver disease, for which only equivocal data are still available [3]. 

The main active components of ginger are gingerols, phenolic ketones with a range of unbranched alkyl chain lengths. They are named according to the length of the chain, as 6-, 8-, 10-, and 12-gingerols [10]. Typically, 6-gingerol is the most abundant active ingredient, while the relative percentage of other gingerols varies slightly with the genetic background of the plant, the climate conditions, and time of harvest [11]. The antioxidant action of 6-gingerol was demonstrated in vitro by DPPH [12] and peroxyl radical scavenging assays [13], and by its ability to lower nitric oxide production in LPS-stimulated mouse macrophages of the J774.1 cell line [14]. Furthermore, 6-gingerol has anti-inflammatory activity, as demonstrated both in vitro and in vivo through the downregulation of pro-inflammatory enzymes, such as leukotriene A4 hydrolase (LTA_4_H) [15] and cyclooxygenase 2 (COX-2) [16]. Recently, 6-gingerol has also been shown to promote caspase-3 mediated apoptosis in cultured cancer cells [17]. 

**Figure 1:**
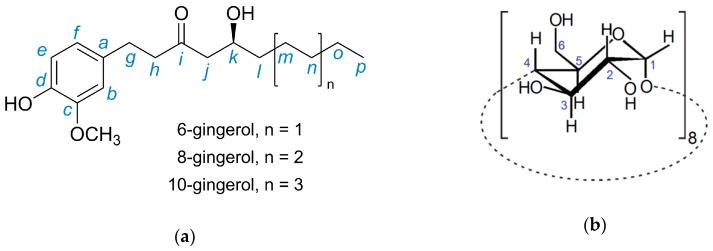
Schematic representation of (**a**) 6-, 8-, and 10-gingerols, the components of the mixture used as guest in this work, and (**b**) the host, γ-CD, depicting the herein adopted atom labeling scheme.

The low solubility of gingerols is associated with low bioavailability from oral ingestion. In this regard, the administration of 250 mg/kg of gingerols to mice was recently shown to afford sub-therapeutic seric concentrations [18]. Possible technological solutions for enhanced solubility and bioavailability comprise the fabrication of nanogingerol [19] and the formulation into microemulsions [20]. In this work, we resort to molecular encapsulation of gingerols with gamma cyclodextrin (γ-CD). The host and the guest are shown in Figure 1. This macrocyclic host is approved for human use without restrictions [21], also being the most digestible of native cyclodextrins [22]. The strategy of forming inclusion compounds with cyclodextrins is frequently employed to improve the aqueous solubility of apolar organic compounds. Nevertheless, out of the countless reports or CD inclusion compounds, those having pungent compounds as the guest are, to the best of our knowledge, under a dozen. Among them, piperine, which is the pungent component of black pepper, has been complexed with native CDs (α-CD, β-CD, and γ-CD) [23,24], and capsaicin from chilies has been complexed by hydroxypropyl-β-CD [25]. Also reported are complexes of allyl isothiocyanate, the pungent compound from wasabi, with the native hosts α-CD and β-CD [26,27,28] and with randomly methylated β-CD (RAMEB) [29], as well as β-CD complexes with benzyl- and phenylethyl isothiocyanates [30].

The combination of fresh ginger juice with dairy products is a growing trend, not only in domestic uses, such as salad dressings and desserts, but also in some marketed products. An example is the ‘Moosa’ yogurt with orange and ginger juices, which were released in the USA in early 2017 [31]. Yogurt fortification with small amounts of dry ginger (0.5 to 2%) was enough to convey superior antioxidant properties and increased shelf-life [32]. Yogurt butter (yayik) that is fortified with 0.5% fresh ginger juice also exhibits a superior shelf-life [33]. In yogurt, the addition of fresh ginger juice is well accepted by tasters in concentrations of up to 4%. In turn, the addition of ginger juice in concentrations of 6 to 10% is not well tolerated, which is mainly due to the strong spicy flavor. Furthermore, it leads to a loss of viability of the fermenting lactic acid bacteria, with consequent alterations to pH, viscosity, and hardness [34]. Yogurt fortification with the pure gingerols fraction of fresh ginger is, to our best knowledge, unprecedented, but advantageous from the nutraceutical viewpoint, as it allows for a precise quantification of actives in the final product. In the present work, two batches of yogurt, one containing the pure gingerols and another containing the γ-CD·gingerols inclusion compound, are prepared and evaluated regarding color, antioxidant properties, and pH stability over three weeks.

## 2. Experimental

### 2.1. Materials

Fresh ginger rhizome was acquired in a local retailer. Pharmaceutical-grade γ-CD (Cavamax W8 Pharma) from Wacker-Chemie was kindly donated by Ashland Specialty Ingredients (Düsseldorf, Germany). Soybean 5-lipoxigenase (freeze-dried powder, ≥50,000 units/mg), 2,2′-azino-bis(3-ethylbenzothiazoline-6-sulphonic acid) (ABTS), linolenic acid (≥99%), and sodium nitroprussiate (≥99%) were acquired from Merk (former Sigma–Aldrich (Madrid, Spain). Trolox, or 6-hydroxy-2,5,7,8-tetramethylchromano-2-carboxylic acid (97% pure) was purchased from ACROS Organics (a subsidiary of Thermo-Fisher, Geel – Belgium). The Griess reagent was prepared previous to use (1% sulphanilamide, 0.1% *N*-1-napthylethylenediamine dichloride, and 3% phosphoric acid). All of the solvents were of analytical grade, except otherwise specified. 

For the production of yogurt, milk with 3.6% fat from Mimosa^®^ and yogurt containing live lactobacilli (Activia^®^ by Danone^®^) were acquired in a local supermarket.

### 2.2. Equipment

Elemental analysis was carried out at the Chemistry Department of the University of Aveiro (by M. Marques) on a CHNS TruSpec^®^ Micro elemental analyser (LECO, St Joseph, MI, USA).

The GC-MS data were collected on a QP2010 Ultra that was equipped with autosampler AOC 20i (Shimadzu, Wemmel, Belgium), ion source of electronic impact, an High-performance Quadrupole Mass Filter, and a Agilent DB-5 ms column with 30 m length, 0.25 mm diameter, and 0.25 µm thickness. The initial temperature of the oven was 50 °C and the injector temperature was 250 °C. The injector went into split mode (1:50). The run was at 69.4 kPa, with a total flow of 65.0 mL/min. and a column flow of 1.22 mL/min. The linear velocity was 40.0 cm/s and the purge flow was 3.0 mL/min. Starting temperature of 50 °C was kept for 3 min., rising at 2 °C per minute to 250 °C, and remaining at that temperature for 10 min. The samples were dissolved in chloroform.

Mass spectrometry (ESI-MS) was performed on a Micromass QTOF-2™ mass spectrometer (Waters, UK). The sample was ionized by electrospray in positive mode, with 3 kilovolts in the needle and 30 volts in the cone.

Laboratory powder XRD data were collected at ambient temperature on an Empyrean PANalytical diffractometer (Cu Kα_1,2_ X-radiation, λ_1_ = 1.540598 Å; λ_2_ = 1.544426 Å) (Bruker AXS, Karlsruhe, Germany), which was equipped with an PIXcel 1D detector and with the sealed tube operating at 45 kV and 40 mA. The data were collected in transmission mode while using the provided Empyrean reflection-transmission spinner by positioning the samples in-between two transparent acetate foils purchased from STOE & Cie GmbH (Darmstadt, Germany), so to ensure the lowest possible background. Intensity data were collected by the step-counting method (step 0.01°), in continuous mode, in the ca. 3.5 ≤ 2θ ≤ 100° range.

Solution-phase ^1^H and ^13^C nuclear magnetic resonance (NMR) spectra were recorded on a Avance 300 spectrometer (Bruker Biospin, Rheinstetten, Germany) at 300.13 and 75.47 MHz, respectively, at room temperature. Deuterated chloroform (CDCl_3_) was used as solvent (^1^H δ 7.26 ppm and ^13^C δ 77.03 ppm) and tetramethylsilane (TMS) as internal reference. The chemical shifts are quoted in parts per million (ppm) and the coupling constants (J) in Hertz (Hz).

^13^C{^1^H} CP/MAS NMR spectra were recorded at 100.62 MHz on a (9.4 T) Avance III 400 spectrometer (Bruker Biospin), with an optimised π/2 pulse for ^1^H of 4.5 μs, 3 ms contact time, a spinning rate of 12 kHz, and 4 s recycle delays. The chemical shifts are quoted in parts per million from tetramethylsilane.

Infrared spectra were obtained as KBr pellets in a 7000 FTIR spectrometer (Mattson, Oakland, CA, USA) (resolution 2.0 cm^−1^; 64 scans per spectrum).

TGA studies were performed on a Shimadzu TGA-50 thermogravimetric analyser (Kyoto, Japan), using a heating rate of 5 °C min^−1^, under air atmosphere, with a flow rate of 20 mL min.^−1^. The sample holder was a 5 mm ø platinum plate and the sample mass was about 5 mg. 

Differential Scanning Calorimetry (DSC) analyses were performed in a power-compensated PerkinElmer Diamond DSC (Llantrisant, UK). Following a five-minute lat period at 20 °C, the samples were heated to 270 °C at a rate of 5 °C/min. The sample mass was of 2.653 mg for ginger extract and 3.616 mg for the respective inclusion complex.

Absorbance readings for the biochemical assays were collected on 96 well quartz plates in a µQuant™ Microplate spectrophotometer (BioTek, Winooski, VT, USA), or in quartz cuvettes in a Shimadzu UVmini-1240 UV-Vis spectrophotometer (Kyoto, Japan). The working wavelengths were 234 nm for the 5-LOX inhibition assay, 470 nm for the β carotene assay, 562 nm for the NO assay, and 734 nm for the ABTS assay.

The color of plain and fortified yogurt was analyzed while using a CM2300d spectrometer with Spectramagic NX software, both from Konica Minolta (Tokyo, Japan).

The pH of yogurt was measured at 20 °C using a Crison Instruments model 5014 pH glass electrode (Barcelona, Spain).

### 2.3. Extraction of Gingerols from Fresh Ginger

600 g of fresh ginger rhizome were finely grated with a rotor and allowed to macerate in isopropanone (500 mL), with stirring at 90 rpm for 48 h [35]. The supernatant was filtered and freeze-dried for another 48 h to obtain the rough extract as a paste. The extract was subject to column chromatography in 60 Å mesh silica while using a mixture of hexane and ethyl acetate with increasing polarity (Table 1).

GC-MS analyses of each fraction allowed identifying the presence of gingerols in fractions 3 and 4. These two were combined and subject to a second column chromatography with hexane and ethyl acetate in varying proportions, collecting fractions of 20 mL (Table 2). Gingerols were identified in fractions 27 to 37.

Further purification was carried out by preparative TLC, in which the sample was diluted with dichloromethane and eluted with dicloromethane/methanol 95:5 *v*/*v*. The solvent was subsequently evaporated under reduced pressure to afford 289 mg of gingerols. Thus, the yield in gingerols regarding the fresh ginger rhizome is of *c.a.* 0.05%. The confirmation of sample composition and purity was carried out by ESI-MS and ^1^H NMR (see details in the Electronic Supporting Information).

ESI^+^-MS (Isopropanone) *m*/*z* (relative intensity %): 371 ([6-gingerol·Na]^+^, 100); 345 ([8-gingerol·Na]^+^, 35); 373 ([10-gingerol·Na]^+^, 47.5). 

^1^H-NMR (300 MHz, CDCl_3_, ppm): *δ* = 0.86–0.91 (3H, *t*, C*_p_*), 1.26–1.39 (8.7H, *m*, C*_l,m,n,o_*), 2.44–2.61 (2H, *m*, C*_j_*), 2.70–2.89 (4H, *m*, C*_g,h_*), 2.94 (1H, *s*, H*_k_*), 3.87 (3H, *s*, O–C*H*_3_), 4.04 (1H, *s*, Ck-O*H*), 5.50 (1H, *s*, C*_d_*-O*H*), 6.64–6.68 (2H, *d*, C*_e,f_*), 6.81–6.84 (1H, *d*, H*_b_*) ppm (for atom labelling, refer to the Figure 1).

^13^C-NMR (500 MHz, CDCl_3,_ ppm): *δ* = 211.5 (C=O), 146.4 (C*_c_*), 144.0 (C*_d_*), 132.6 (C*_a_*), 120.7 (C*_f_*), 114.4 (C*_e_*), 111.0 (C*_b_*), 67.7 (O-*C*H_3_), 55.9 (C*_k_*), 49.3 (C*_j_*), 45.4 (C*_h_*), 36.4 (C*_l_*), 31.7 (C*_g_*), 29.3 (C*_n_*), 25.1 (C*_m_*), 22.6 (C*_o_*), 14.0 (C*_p_*) ppm.

FTIR (ν, cm^−1^) = 3461vs, 2954 vs, 2951 vs, 2942 sh, 2937 sh, 2929 vs, 2926 sh, 2921 sh, 2918 sh, 2884 s, 2876 sh, 2870 sh, 2857 s, 1736 w, 1707 s, 1704 s, 1698 s, 1693 s, 1681 m, 1671 sh, 1665 w, 1659 w, 1649 w, 1643 sh, 1632 sh, 1619 sh, 1610 sh, 1602 m, 1594 sh, 1573 w, 1556 w, 1562 w, 1555 sh, 1551 sh, 1547 w, 1515 vs, 1493 w, 1464 s, 1453 s, 1439 sh, 1431 s, 1407 m, 1383 sh, 1375 sh, 1370 s, 1359 sh, 1271 vs, 1236 s, 1208 s, 1187 sh, 1152 s, 1131 sh, 1123 s, 1087 m, 1034 s, 992 sh, 927 w, 894 w, 853 w, 824 sh, 813 m, 796 m, 724 w, 706 w, 671 w, 665 sh, 624 w, 557 m.

### 2.4. Preparation of γ-CD·Gingerols

To a solution of γ-CD (632 mg 0.445 mmol) in water (1.5 mL) at 40 °C was added a solution of gingerols (140 mg, 0.445 mmol) in ethanol (1.5 mL) dropwise. The mixed solution was allowed to stir 18 h at 40 °C and then slowly cooled to form a precipitate that was isolated by centrifugation at 3500 revolutions per minute (rmp) for 50 min. The product was stored in a desiccator overnight before it was weighed. Yield: 721.5 mg, 93%).

Elemental anal. calc for (C_48_H_80_O_40_)·0.54(C_17_H_26_O_4_)·0.19(C_19_H_30_O_4_)·0.27(C_21_H_34_O_4_)·14.4H_2_O (1871.4): C, 42.65; H, 7.417; obtained C, 42.65; H, 7.298.

FTIR ν (tilde, cm^−1^) = 3366 vs, 2946 sh, 2926 m, 2902 sh, 2851 sh, 1719 w, 1707 w, 1703 w, 1698 m, 1693 m, 1687 sh, 1681 sh, 1677 w, 1672 w, 1664 sh, 1659 sh, 1657 w, 1649 w, 1643 sh, 1632 w, 1625 sh, 1620 w, 1613 w, 1602 m, 1579 sh, 1572 w, 1565 w, 1563 w, 1555 w, 1551 w, 1547 w, 1543 sh, 1535 sh, 1529 sh, 1524 sh, 1516 m, 1503 w, 1492 sh, 1468 sh, 1460 w, 1453 w, 1431 sh, 1421 m, 1415 m, 1383 m, 1374 sh, 1351 sh, 1336 m, 1300 w, 1273 m, 1255 w, 1240 m, 1200 w, 1158 s, 1126 sh, 1105 m, 1079 s, 1051 sh, 1026 vs, 1001 s, 942 m, 935 sh, 917 sh, 890 w, 870 sh, 860 w, 810 sh, 793 sh, 761 w, 704 m, 608 m, 580 m, 554 sh, 529 m, 476 w, 449 sh, 442 sh, 412 w, 400 sh, 390 w, 362 w, 358 w, 346 w, 338 sh, and 331 w.

^13^C-NMR CP/MAS (12 kHz, 25 °C, ppm): δ = 210.0 (guest, C=O), 147.5 (guest, C*_c_*), 144.6 (guest, C*_d_*), 132.5 (guest, C*_a_*), 121.0, (guest, C*_f_*), 115.4 (guest, C*_e_*), 111.7 (guest, C*_b_*), 105.2, 104.1 (γ-CD, C_1_), 82.9, 82.6, 82.3, 81.1 (γ-CD, C_4_), 74.6, 74.1, 73.7, 73.2, 73.0, 72.6, 71.9, 71.2 (γ-CD, C_2,3,5_), 67.5 (guest, O-*C*H_3_), 60.8, 60.4 2 (γ-CD, C_6_), 56.7 (guest, C*_k_*), 50.0 (guest, C*_j,h_*), 36.7 (guest, C*_l_*), 31.7 (guest, C*_g_*), 29.2 (guest, C*_n_*), 25.0 (guest, C*_m_*), 22.5 (guest, C*_o_*), and 14.2 (guest, C*_p_*).

### 2.5. Biochemical Assays

The activity of gingerols and γ-cyclodextrin·gingerols, both in their free form and from yogurt extracts, was evaluated by a series of in vitro assays. All of the determinations were carried out in three independent assays.

#### 2.5.1. ABTS^•+^ Discoloration Assay

This method was performed according to the procedure that was described by Wali et al. [36]. Briefly, the ABTS^•+^ solution was prepared by reacting the stock solution of ABTS (7 mM) with potassium persulfate (2.45 mM) in a ratio of 1:1. The solution was allowed to stand in the dark at room temperature for 12–16 h. Before use, the stock solution was diluted with ethanol to get an absorbance of 0.70 ± 0.020 at 734 nm. Several concentrations of sample extracts/standard were dissolved in 250 µL of diluted ABTS^•+^ solution. Absorbance values (734 nm) were read after 6 min. of incubation and the percentage of inhibition was calculated while using the Equation (1):

(1) $$ABTS^{•+}\begin{array}{ccc} scavenging & activity & (\%)=\frac{Abs_{control}-Abs_{sample}}{Abs_{control}} \end{array}\times 100$$

In the Equation (1), Abs*_control_* is the absorbance of ABTS radical the control without extract addition and Abs*_sample_* is the absorbance of ABTS radical with extract. 

The results were expressed as IC_50_ (concentration of the extract able to inhibit the 50% of the ABTS^•+^) of each extract. Ascorbic acid and trolox were used as the positive controls.

#### 2.5.2. β-Carotene Bleaching Assay

The assay was performed, as described by Afonso et al. [37]. Briefly, from a solution of 20 mg of β-carotene in 10 mL of chloroform, a volume of 1 mL was retrieved and mixed with 1 g of tween-80; chloroform was removed by evaporation and the mixture was added with 50 mg of linoleic acid and 100 mL of distilled water, and then homogenised to obtain a stock emulsion of β-carotene/linoleic acid. 250 µL aliquots of the stock emulsion were mixed with 50 µL of sample at different concentrations (ranging from 38.6 to 102 μM) and the absorbance at t = 0 was immediately recorded. After incubation at 50 °C for 2 h, the reaction was stopped while using an ice bath and absorbance values were measured once more. The % of inhibition was calculated while using the Formula (2):

(2) $$\%Inhibition=\frac{\left(C_{t0}-C_{tf}\right)-\left(S_{t0}-S_{tf}\right)}{C_{t0}-C_{tf}}\times 100$$

C*_t_*_0_ and C*_t_*_2_ are the absorbance of the control at *t* = 0 min., and *t* = 120 min., respectively, and S*_t_*_0_ and S*_tf_* are the absorbance values of the sample at *t* = 0 min. and *t* = 120 min., respectively. BHA was used as the positive control and chloroform without β-carotene was used as blank. Note that the gingerols samples were previously dissolved in ethanol, since they are not soluble in water.

#### 2.5.3. Chemical NO Scavenging Assay 

This assay was adapted from the procedures that were previously described by Catarino et al. [38]. The stock solution comprised sodium nitroprusside (1 g/L, 3.82 mM), γ-CD (9 g/L, 6.32 mM), and ascorbic acid (1 g/L, 5.66 mM) in phosphate buffer and gingerols (0.98 g/L, 3.16 mM) in ethanol. In each assay, 50 µL of sample or control were mixed with 50 μL of sodium nitroprusside and 100 μL of appropriate solvent to attain a working concentration of 1 mM and a final volume of 200 μL (1:1 mixture of ethanol and aqueous phosphate buffer). The samples were subject to irradiation with light for 10 min. at ambient temperature and subsequently added with 100 µL of Griess reagent. Colour control was treated with 100 µL of a 1% phosphoric acid solution. The samples were further allowed to react in the absence of light before measuring the absorbance. The IC_50_ value for the NO scavenging activity was determined by plotting the percentage of inhibition of nitrite generation in the presence of gingerols and γ-CD against the tested concentrations.

#### 2.5.4. Soybean 5-Lipoxigenase (5-LOX) Assay 

5-LOX assay was performed while using linoleic acid as a substrate, as previously described by Catarino et al. [38]. Linoleic acid (500 µM) was prepared by dilution of a stock solution (1M) in 0.2 M borate buffer (pH 9.0) containing 0.05% (*v*/*v*) tween-20. Test solutions of gingerols, γ-CD, γ-CD·gingerols were prepared at the concentrations of 0.3, 0.6, 1.0, 1.5, 2.0, 3.0 and 4.0 mM in a 1:1 mixed solution of DMSO and PBB/tween-20. Ascorbic acid was used as control or reference substance, in concentrations of 0.1, 0.2, 0.4, 0.7, 1.0, 1.5 and 2.0 mM, also in the same solvent mixture. Assays were performed on a 96-well quartz plate by combining 12.5 units of 5-LOX with 25 µL of each sample dilution and incubating over 10 min. at 37 °C. The reaction was initiated by the addition of 50 µL of linoleic acid and the plate was immediately placed in an UV/vis plate reader to read absorbance. Values were recorded every 60 s for a total time of 600 s (10 min.), at 234 nm. The appearance of a conjugated diene promotes an increase in the absorbance that is proportional to the reaction time as the linoleic acid is converted to 1–3-hydroperoxylinoleic acid, thus generating a curve. The value for the inhibitory percentage of the enzyme activity was calculated while using Formula (3):

(3) $$\%Inbitition=\frac{m_{A}{{}_{c}}_{0}-m_{Aet}}{m_{Ac0}}\times 100$$

In Formula (3), *m_Ac_*_0_ is the slope of the straight line portion of the curve that is generated by the negative control and *m_Aet_* the slope of the straight line portion of the curve generated by each sample. 

### 2.6. Formulation of Yogurts Fortified with Gingerols and γ-Cyclodextrin·Gingerols

Three different varieties of yogurt were prepared—plain yogurt, gingerols yogurt, and γ-CD·gingerols yogurt. Each of these batches comprised three samples, making a total of nine samples. The yogurts were produced in the laboratory, while using UHT milk with 3.6% fat and commercial yogurt as the source of bacterial culture. Each vial, containing 5 mL of milk, was inoculated with c.a. 0.6 g (12%) of commercial yogurt starter culture containing *Lactobacillus bulgaricus*, *Streptococcus thermophilus*, and *Bifidobacterium animalis DN 173.010*. After mixing the two ingredients, the fortificant was added, comprising 0.038 g (0.75%) of gingerols or 0.050 g (1%) of γ-CD·gingerols. The nine vials were kept at 40 °C for 18 h in a thermostatic water bath, being subsequently removed and stored in the refrigerator. 

### 2.7. Analysis of Fortified Yogurts

#### 2.7.1. Colour Measurement

Each sample was placed on a watchglass, with the spectrometer probe over it. A small distance was kept between the sensor and the sample, to avoid contamination. This small distance, associated with the fact that the surface of the yogurt is not completely flat, induced some variability in the measured luminosity, i.e., the parameter L^*^ has greater variation. Five or 10 replicates were collected for each sample in order to reduce these sources of error (n = {5, 10}). Table 3 lists the values of the parameters L*, a* and b*.

ΔL^*^, Δa^*^, and Δb^*^ were obtained by subtracting the values of ȳ (L^*^), ȳ (a^*^), and ȳ (b^*^) of fortified yogurt to those of the reference (plain yogurt) and then used to calculate the associated error (ΔE) between the reference and the innovative samples (fortified yogurt) by applying Formula (4).

(4) $$\Delta E=\sqrt{{\left(\Delta L*\right)}^{2}+{\left(\Delta a*\right)}^{2}+{\left(\Delta b*\right)}^{2}}$$

#### 2.7.2. Yogurt Antioxidant Capacity

The antioxidant activity of the plain and fortified yogurts (with gingerols and γ-CD·gingerols) were estimated by the ABTS method (as described in 2.5.1.) and expressing the results as equivalents of ascorbic acid (EAA). For this, extracts were prepared by treating 1 g of yogurt with 1 mL of ethanol, followed by centrifugation and the collection of the supernatant.

#### 2.7.3. Yogurt pH Over Time

The pH of yogurt (both plain and fortified) was monitored over a period of eight weeks, with a weekly periodicity for the first four weeks and monthly periodicity after that. Following proper calibration, measurements were made by simple immersion of the electrode in each yogurt vial. The results are the average of three readings for each of the three independent samples in each batch.

## 3. Results and Discussion

### 3.1. Isolation of Gingerols

In the present work, gingerols were obtained from fresh ginger rhizome as the raw material, keeping in mind the minimisation of time, energy, and the heat-induced degradation of the gingerols that are associated with traditional drying procedures. Yet, the extraction from fresh ginger rhizome results in a crude extract that contains a high amount of water, requiring further purification. Hence, after the maceration of grated ginger rhizome for 48 h, the crude extract was subjected to chromatographic purification to isolate the gingerols. Subsequent characterisation with analytical techniques confirmed the purity of the sample. Moreover, results showed that the gingerols were sufficiently free of organic solvents to be tested in food matrices.

**Figure 2:**
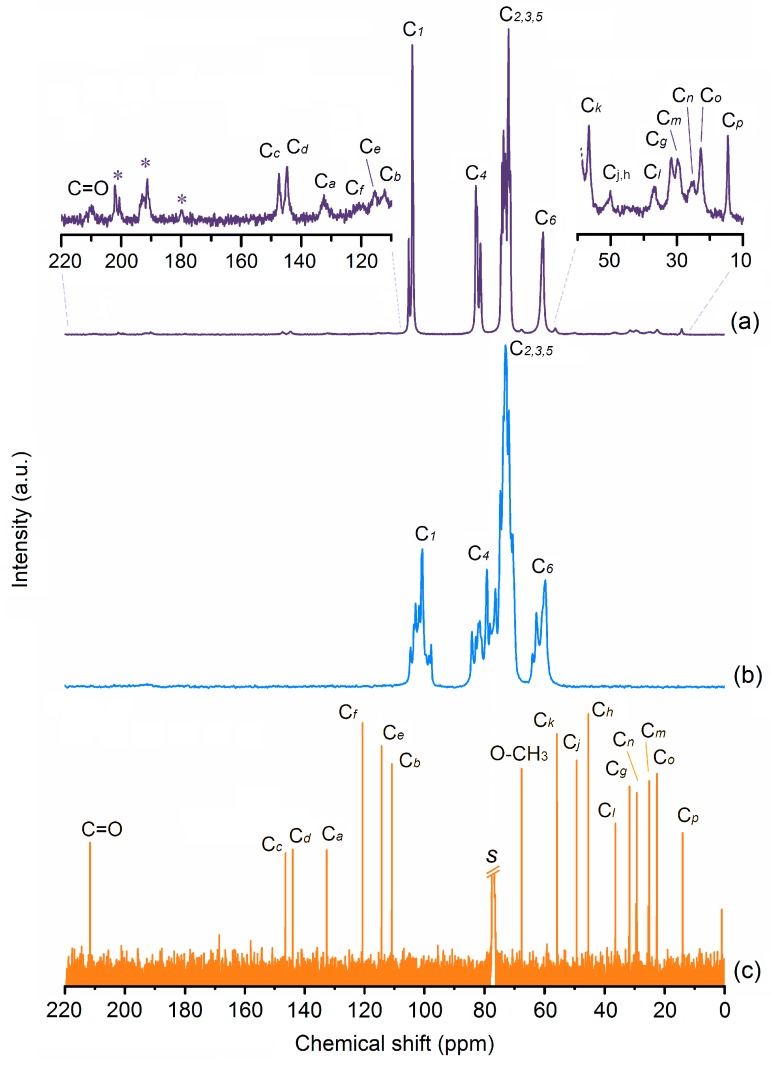
^13^C{^1^H} CP/MAS NMR spectra for (**a**) γ-CD∙gingerols, (**b**) the γ-CD host and solution-phase ^13^C NMR spectrum of gingerols (**c**). The insets show expansions of the 220-110 and 60-10 ppm regions of the spectrum of γ-CD∙gingerols, where guest resonances can be observed. Spinning sidebands in ^13^C{^1^H} CP/MAS NMR spectra are denoted by asterisks and the solvent (CDCl_3_) signal in the solution-phase ^13^C NMR spectrum is identified with *s*.

ESI-MS spectrometry was used to quantify the amounts of of 6-, 8-, and 10-gingerols in the sample. These are given by the relative intensities of their mass spectra peaks (see Supporting Information Section S2). A careful analysis allows for learning that the extract is composed of 54.05% of 6-gingerol (C_17_H_26_O_4_), 19.45% of 8-gingerol (C_19_H_30_O_4_) and 26.5% of 10-gingerol (C_21_H_34_O_4_), thus having a pondered molecular mass of 314.71 g/mol. We also note that ^1^H NMR (Figure S1 in the Supporting Information) and ^13^C NMR (Figure 2c) spectra are coherent with those previously reported for gingerols [39,40], thus confirming the identification of these compounds.

### 3.2. Preparation and Characterisation of γ-CD·Gingerols

The preparation of the γ-CD·gingerols inclusion complex was carried out by the co-dissolution of equimolar amounts of host and guest, followed by co-precipitation. The solubilisation of the guest (gingerols) was carried out in ethanol, a non-toxic and environmentally friendly solvent, so that the inclusion compound is adequate for human consumption.

A first insight into the product of inclusion, hereafter denominated as γ-CD·gingerols, can be obtained by examining the FT-IR spectrum of this sample in comparison to those of its components. Vibrational bands of the host, also featuring a few bands assigned to the gingerols, largely dominate the spectrum of γ-CD·gingerols. These bands of the guest appear mostly unshifted, thus indicating that the structural integrity of the gingerols was preserved during the inclusion process. Moreover, the purity of the γ-CD·gingerols and the inclusion stoichiometry were confirmed by elemental analysis. Data (listed in Section 2.4) shows that the 1:1 stoichiometry used in the starting mixture for the inclusion was retained, i.e., the host-to-guest proportion in γ-CD·gingerols is of 1:1. The empirical formula is calculated as C_48_H_80_O_40_·[0.54C_17_H_26_O_4_·0.19C_19_H_30_O_4_·0.27C_21_H_34_O_4_]·14.4H_2_O. Note that, since cyclodextrin molecules may stack as dimers, a 1:1 proportion can correspond, in practical terms, to a 2:2 stoichiometry for the γ-CD·gingerols inclusion compound. Also noteworthy, the number of hydration water molecules *per* host unit is more than twice of that occurring in pure γ-CD heptahydrate, a feature that is typically associated with γ-CD inclusion compounds [41]. This is discussed with further detail in Section 3.2.2.

#### 3.2.1. ^13^C-NMR

Figure 2 depicts the solid-state ^13^C{^1^H} CP-MAS NMR spectra of γ-CD hydrate and γ-CD∙gingerols, in comparison with the solution-phase ^13^C spectrum of the pure gingerols extract in CDCl_3_ (see carbon labelling of gingerols and γ-CD in Figure 1). 

The carbon resonances of the host change from multiple signals in the spectrum of pure γ-CD hydrate [42,43] to very sharp and narrow signals in the spectrum of γ-CD·gingerols. In the case of C1 and C6, one main signal with a shoulder or a smaller resonance are observed, indicating symmetrisation of geometrical parameters for these carbons. This observation is consistent with the formation of supramolecular structures with channel packing, which is typical of γ-CD inclusion compounds. There is also significant narrowing of the resonances of C4 and C2,3,5 with a strong reduction of the fwhm (full width at half maximum).

The guest signals are also present, albeit with a lower intensity than those of the host. For better identification, the two main regions where these signals occur are expanded and presented as insets where all carbons of the gingerols are duly identified, with exception of that of the methoxyl group that is observed between the C2,3,5 and the C6 signals of γ-CD.

#### 3.2.2. Powder X-Ray Diffraction

**Figure 3:**
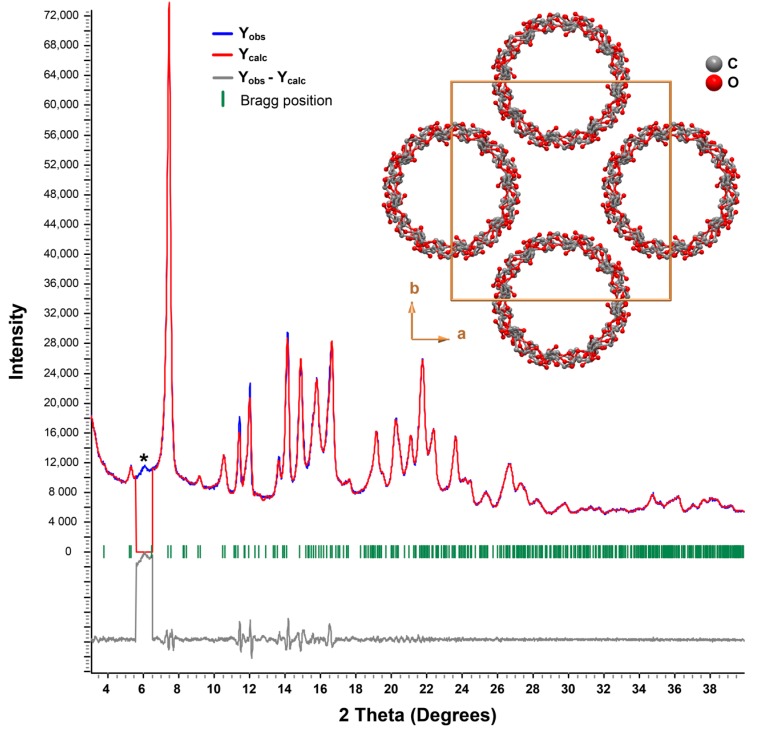
Final Pawley plot of γ-CD·gingerols. Observed data points are indicated as a blue line, the best fit profile (upper trace) and the difference pattern (lower trace) are drawn as solid red and grey lines, respectively. Green vertical bars indicate the angular positions of the allowed Bragg reflections. The inset depicts a perspective view along the [001] direction of the close packing of γ-CD in crystal structures pertaining inclusion compounds. The asterisk depicts the presence of a small amount of an unidentified crystalline phase.

An initial short-time powder X-ray diffractogram (PXRD) of γ-CD·gingerols depicts a crystalline material with some traces of amorphous background, denoted by a halo of diffraction in the baseline (not shown). For better intensity data, a second PXRD of the same sample was collected overnight (see details in Section 2.2). It is represented by the blue line in Figure 3.

The collected powder X-ray diffraction pattern was indexed while using the LSI-Index algorithm that was implemented in TOPAS-Academic V5 [44], and a whole-powder-pattern Pawley fit permitted to unequivocally confirm one again the cubic *P*42_1_2 space group as the most suitable. A modified Thompson-Cox-Hastings pseudo-Voigt (TCHZ) profile function was selected to generate the line shapes of the simulated diffraction peaks [45]. A Pawley refinement of the collected PXRD led to refined tetragonal unit cell parameters of *a* = *b* = 23.885(4) Å, *c* = 23.351(4) Å, *α* = *β* = *γ* = 90°, ultimately confirming the formation a new inclusion compound (Figure 3) [46]. Although no contamination of pure γ-CD heptahydrate is presented, one notes the existence of a small reflection at low angles attributable to an unidentified (poorly) crystalline phase (asterisk in Figure 3). The unit cell parameters determined for γ-CD∙gingerols are, thus, characteristic of γ-CD inclusion compounds. So far, all known structures with this host are isotypical, crystallising in tetragonal space group *P*42_1_2. The packing arrangement has a high symmetry, with all of the γ-CD∙guest units being stacked in the form of infinite channels, as depicted in the inset of Figure 3. The longitudinal axes of the channels (parallel to the [001] direction of the unit cell) are aligned to form squares, a geometry that results in the formation of wide inter-channel void spaces that are able to retain a large amount of water molecules (not shown in Figure 3 for clarity). Thus, it is fair to assume that the same geometry occurs in γ-CD∙gingerols, and that it accounts for the increase in hydration waters that was observed in elemental analysis. 

#### 3.2.3. Differential Scanning Calorimetry (DSC)

**Figure 4:**
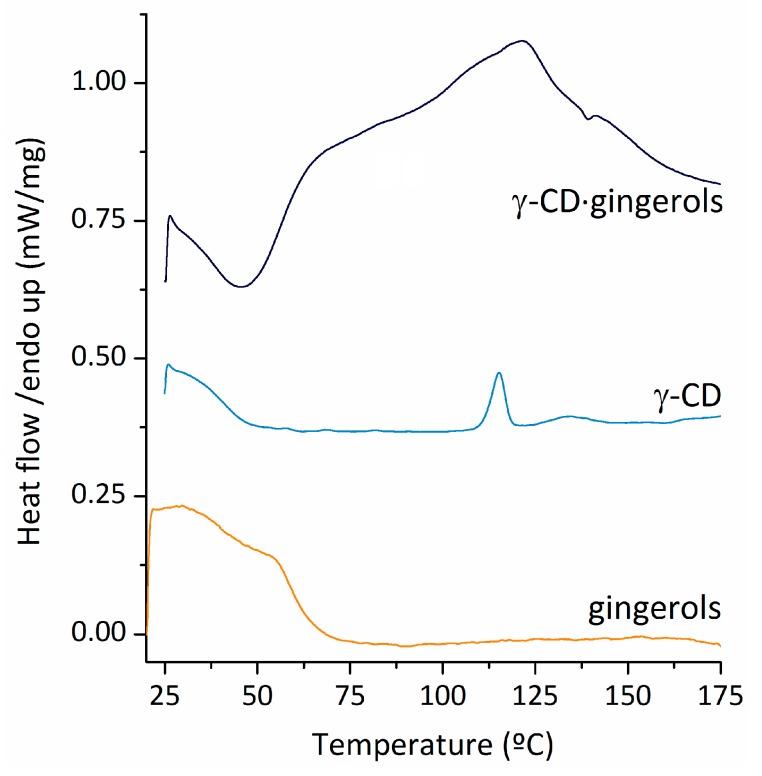
Differential scanning calorimetry traces of gingerols (**bottom**), pure γ-CD heptahydrate (**middle**) and γ-CD·gingerols (**top**).

The DSC trace of the gingerols, as depicted in Figure 4, shows a broad peak centred around 29.5 °C that can be associated with the melting temperature of 6-gingerol [47] and 10-gingerol [48]. The broad aspect of the peak is associated with the amorphous nature of the sample [47]. A second exothermal event is observed, peaking at around 54 degrees. For pure γ-CD heptahydrate, DSC shows two peaks associated with dehydration, a sharp one at 115 °C, and a broad and less intense one around 130 °C.

A significant alteration of the DSC profile is observed in the sample of γ-CD∙gingerols. The thermal events associated with melting of gingerols are not observed in the corresponding temperature interval, and instead two partially overlapped thermal events are visible, the first having a maximum at 68–70 °C and a second peaking at 125 °C. The absence of the melting peak for gingerols shows that each one of these molecules is separated from the next by inclusion into the cavity of γ-CD. The two thermal events that are observed are in the DSC trace of γ-CD∙gingerols are most likely associated with loss of hydration waters, known to exist in higher amounts in the inclusion compound, and occurring over a broader temperature range.

### 3.3. Antioxidant and Anti-Inflammatory Studies

The effect of γ-CD on the antioxidant activity of gingerols is evaluated by the ABTS^•+^ and beta-carotene assays, while NO^•^ scavenging and 5-LOX inhibition assays are employed to evaluate the possible differences associated to their anti-inflammatory abilities. Table 4 presents the results.

Gingerols and γ-CD·gingerols both feature ABTS^•+^ scavenging activity values that are very close to that of the standard, trolox. Note that the EC_50_ values of the two known substances used as positive control in this method, were similar to literature ones: the EC_50_ of trolox is similar to that previously described by Lin et al. (7.36 ± 0.43 µM) [49], and the EC_50_ of ascorbic acid is in good agreement with the value that was reported by Elosta et al. (19.5 ± 0.3 µM equivalents of trolox) [50], which validates the method herein employed. The activity of the gingerols extract obtained herein is a bit low in comparison with that of pure 6-gingerol, which was reported by Lin et al. to have an EC_50_ value of 2.53 ± 0.07 µM [49]. Our result can be attributed to the presence of 8-gingerol and 10-gingerol in the gingerols extract, which are known to have lower activity in the ABTS^•+^ assay in regard to 6-gingerol [51].

The EC_50_ values for free and encapsulated gingerols were very similar in the β-carotene method (83 ± 5 μM and 85 ± 7 μM, respectively), hence indicating that the presence of γ-CD does not alter gingerols antioxidant efficacy, particularly in what concerns their ability to prevent lipid peroxidation. In comparison with the standard, BHA (*tert*-butyl-4-hydroxyanisole), gingerols, and γ-CD·gingerols were roughly twelve times less effective. 

Regarding NO^•^ scavenging, the sample of γ-CD·gingerols featured a slightly higher activity than pure gingerols, which was about half that of the ascorbic acid. Moreover, with respect to this standard, gingerols and γ-CD·gingerols showed only 2–3 times less potency against LOX. Thus, it can be concluded that gingerols might feature a dual action in towards inflammatory processes, both by direct interaction, as indicated by our results, and by the inhibition of its production in immune competent cells, as reported in the literature [14,52,53,54]. 

The results that are presented in this section indicate that γ-CD is an adequate carrier for the gingerols extract, as it does not interfere with its effectiveness or, in a few instances, contributes to its slight increase.

### 3.4. Application of γ-CD·Gingerols in Yogurt

Yogurt is a food with high nutritional quality and a soft texture, being adequate for consumption across all age groups. These qualities make yogurt an excellent candidate for conveying natural compounds with nutraceutical properties, as is the case of gingerols. In the present study, we have prepared two batches of fortified yogurt, one containing gingerols and another containing γ-CD·gingerols. These were added to the mixture of milk and lactic ferments prior to incubation at 40 ºC, for better homogeneity of the final product. 

Homogenising the gingerols extract into the milk and ferment matrix was difficult and somewhat time-consuming. In turn, the homogenization of γ-CD·gingerols was achieved with relative ease. The superior technical performance of the inclusion compound is evidence of the advantages of using cyclodextrin encapsulation in food applications. 

#### 3.4.1. Colourimetry of the Fortified Yogurt

**Figure 5:**
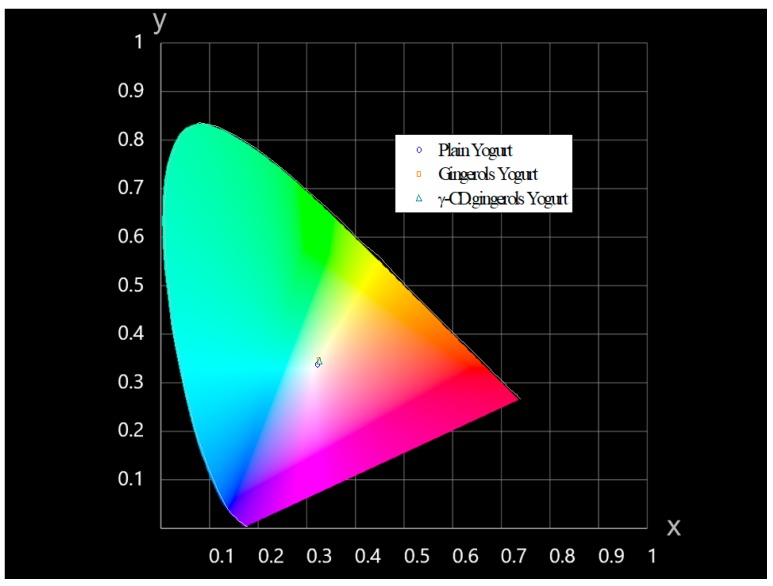
Chromaticity diagram with the representation of the colour coordinates of plain yogurt and fortified yogurt samples. Data corresponds to the mean value of readings of three different samples from each batch.

The colour of yogurt fortified with gingerols and γ-CD·gingerols, as well as plain yogurt (used as reference), was assessed by the colour parameters L*, a* and b*, as measured by reflectance spectrophotometry. The difference in colour is expressed as the associated error or ΔE between the reference sample, i.e., plain yogurt, and the innovative samples (refer to the Experimental section for details on the calculation). The ΔE of the yogurt that was fortified with gingerols was 2.84, while that of yogurt with γ-CD·gingerols was ΔE = 1.94. According to the definition of CIE (Commission internationale de l′éclairage), no differences are noticed when ΔE > 1 and, for 1 > ΔE > 2, only an experienced observer can notice the difference. Based on that, the gathered results indicate that the change of colour of the yogurt that was fortified with γ-CD·gingerols was very subtle, when compared to that of plain yogurt, and that it would be undetected by the average consumer. In turn, any observer would detect changes in the colour of yogurt with the pure gingerols (ΔE > 2). However, it should be mentioned that, even in that case, the difference is not salient, and the colour of gingerol yogurt can be described as a pale beige tone (resembling ‘latte macchiato’). For better visualization, the L*, a*, b* parameters of the three yogurt batches were converted to (x,y) coordinates and graphically represented in a CIE 1931 chromaticity diagram, as depicted in Figure 5.

#### 3.4.2. Antioxidant Properties of Fortified Yogurt

The ability of gingerols and γ-CD·gingerols to retain their antioxidant activity in the yogurt was tested by the ABTS^•+^ scavenging assay on yogurt extracts, each being prepared by centrifuging 1 *g* of yogurt with 1 mL of ethanol (see 2.7.2. for details). The extracts of plain yogurt were also prepared and tested, having a low antioxidant capacity, with a value of 0.826 ± 0.023 mM of equivalents of ascorbic acid (EAA), which was subtracted to that of the fortified yogurts. Note that, while these two yogurts contain approximately the same mass percentage of fortificant (0.75–1%), the high molecular weight of γ-CD·gingerols results in differences in the actual content in gingerols when expressed in molar concentrations (300 for pure gingerols and 100 μM for γ-CD·gingerols, respectively). Because of that, we have normalised the results with respect to the gingerol content. The obtained antioxidant activities, normalised to samples containing 100 μM gingerols, were of 4.40 ± 0.87 mM EAA and 5.59 ± 1.23 mM EAA, for yogurt fortified with gingerols and γ-CD·gingerols, respectively. These results indicate that the inclusion of gingerols into γ-CD inclusion has a favorable effect on the antioxidant action of gingerols in yogurt.

#### 3.4.3. Stability of Yogurt pH Under Storage

The pH of the different yogurt varieties (Table 5) was around 3.7, and it remained unaltered during the first two weeks of storage. Over the next two weeks it lowered very slightly, to approximately 3.5–3.6 after one month of storage, and remained similar after two months of storage. This shows that pH is not the most indicative measurement of yogurt degradation.

The odor of the formulated yogurts was registered concomitantly with the pH measurements. As expected, plain yogurt was the first to develop a foul odor, starting around the third week and gaining intensity over the fourth week. Fortified yogurt remained unaltered in aroma until the eighth week of observation. The most notorious alterations, with the development of a foul odor, occurred in the yogurt with γ-CD·gingerols, most likely because of its lower total gingerols content.

## 4. Conclusions

The inclusion of multi-component natural products, such as botanical extracts and essential oils, into cyclodextrins, is a growing research trend due to the improved solubility and stability brought about by these hosts. Nevertheless, the characterisation of the products is quite challenging due to the complexity that is associated with the presence of various guest molecules inside the CD cavity and the variability in their proportion that might occur during the inclusion process. In the present work, we have resorted to a variety of solid and liquid-state analytical techniques to study the gingerols fraction that was obtained from fresh ginger rhizome and its inclusion compound with γ-CD. The gathered data allowed ascertaining the composition of gingerols as having 54.05% of 6-gingerol, 19.45% of 8-gingerol, and 26.5% of 10-gingerol. Furthermore, we demonstrated the formation of a microcrystalline 1:1 inclusion compound of the gingerols with γ-CD, with a crystalline cell belonging to the tetragonal space group 42_1_2 and having the γ-CD·gingerols units stacked in infinite channels, with water molecules filling the wide inter-channel spaces.

The activity of γ-CD·gingerols in the ABTS^•+^, and β-carotene bleaching assays is similar to that of pure gingerols. For the NO and 5-LOX inhibition assays, measuring the anti-inflammatory activity, γ-CD·gingerols had similar or slightly better performance than gingerols. Thus, the beneficial properties of gingerols are kept upon inclusion into γ-CD, which demonstrates its suitability as a molecular carrier for gingerols. 

The incorporation of γ-CD·gingerols into a mixture of milk and lactic ferment was rapidly and effectively achieved and it resulted in the production of yogurt with a homogeneous aspect and no discernible colour difference with regard to plain yogurt. In comparison with pure gingerols, which were more difficult to homogenously disperse into the yogurt pre-mixture, the inclusion compound has technical advantages. Moreover, the fortification of yogurt with gingerols and γ-CD·gingerols conveys it a superior anti-oxidant activity, which makes this product an innovative nutraceutical.

## Figures and Tables

**Table 1:** Volume and composition of the various solvent fractions used in the first separation of the components existing in the ginger extract.

Fraction Number	Volume (mL)	Composition (*v*/*v*)
1	70	hexane/ethyl acetate 4:1
2	60	
3	20	
4	70	hexane/ethyl acetate 1:1

**Table 2:** Volume and composition of the fractions in the second fractioning of ginger extract.

Fractions	Total Volume (mL)	Composition (*v*/*v*)
1–17	340	hexane/ethyl acetate 5:1
18–27	200	hexane/ethyl acetate 3:2
28–40	260	hexane/ethyl acetate 1:1

**Table 3:** Sample colour parameters, with mean (ȳ) ± standard deviation for each batch.

Sample	*L*	a*	b*	ȳ (L*)	ȳ (a*)	ȳ (b*)
			entry 1		data	data
**Plain Yogurt**						
1 *(n = 5)*	88.980	−1.408	3.500	87.986 ± 5.003	−1.317 ± 0.286	4.086 ± 1.053
2 *(n = 5)*	92.418	−1.548	5.302			
3 *(n = 5)*	82.561	−0.997	3.456			
**Gingerols Yogurt**						
4 *(n = 10)*	90.587	−1.830	6.524	89.076 ± 3.020	−1.874 ± 0.155	6.646 ± 0.344
5 *(n = 10)*	91.044	−2.048	7.035			
6 *(n = 10)*	85.598	−1.746	6.380			
**γ-CD·gingerols Yogurt**						
7 *(n = 5)*	83.964	−1.372	3.678	87.615 ± 5.258	−1.620 ± 0.310	5.968 ± 2.066
8 *(n = 5)*	85.240	−1.520	6.536			
9 *(n = 5)*	93.642	−1.968	7.692			

**Table 4:** Inhibition of 5-LOX and radical scavenging activities of the gingerols in the presence and absence of γ-CD, as compared to those of reference antioxidants.

Compound	ABTS^•+^ EC_50_ (μM)	β-Carotene EC_50_ (μM)	NO^•^ Scavenging ^1^ (%)	5-LOX EC_50_ (μM)
γ-CD	—	> 100 ^2^	11.3 ± 1.5	> 1000 ^3^
gingerols	9.13 ± 1.03	83 ± 5	17.5 ± 3.2	695 ± 47
γ-CD·gingerols	8.80 ± 0.81	85 ± 7	25.3 ± 1.2	629 ± 101
Trolox	7.99 ± 0.99	—	—	—
Ascorbic acid	183.3 ± 14.9	—	50.3 ± 1.9	234 ± 21
BHA	—	7.0 ± 0.1	—	—

^1^ All compounds and the standard were tested at a concentration of 1mM; ^2^ NO inhibition at the highest concentration tested, 100 μM, was of 0%; ^3^ 5-LOX inhibition at 1000 μM was c.a. 42% and no higher concentrations were tested.

**Table 5:** Evolution of the pH of the different yogurt varieties over an observation period of eight weeks.

Batch	Week 1	Week 2	Week 3	Week 4	Week 8
Plain Yogurt	3.71 ± 0.06	3.73 ± 0.02	3.62 ± 0.04	3.59 ± 0.06	3.59 ± 0.05
Gingerols Yogurt	3.72 ± 0.07	3.78 ± 0.06	3.65 ± 0.06	3.60 ± 0.04	3.62 ± 0.09
γ-CD·Gingerols Yogurt	3.65 ± 0.06	3.71 ± 0.01	3.59 ± 0.04	3.56 ± 0.07	3.53 ± 0.02

## Supplementary Materials

The following are available online at https://www.mdpi.com/2218-273X/10/2/344/s1, Section S1, reporting to NMR of the gingerols, contains Figure S1: ^1^H-NMR spectrum of gingerols, Table S1: ^1^H-NMR data of gingerols compared to 6-gingerol from a literature report; section S2 reports MS data for gingerols, contains ginger rhizome extract composition (in gingerols, from MS data) and Figure S2, mass spectrum of the gingerols.
