# Supplementary material for: Solid γ-Cyclodextrin Inclusion Compound with Gingerols, a Multi-Component Guest: Preparation, Properties and Application in Yogurt
Source: Biomolecules. 2020 Feb 22;10(2):344. doi: 10.3390/biom10020344 (PMC7072569; doi:10.3390/biom10020344)

biomolecules

Electronic Supplementary Information

Solid γ-cyclodextrin inclusion compound with gingerols, a multi-component guest: preparation, properties and application in yogurt

Joana M. Pais, Bruna Pereira, Filipe A. Almeida Paz, Susana M. Cardoso, Susana S. Braga

S1. ^{1}H NMR

Figure S1 shows the ^{1}H NMR spectrum of the gingerols sample.

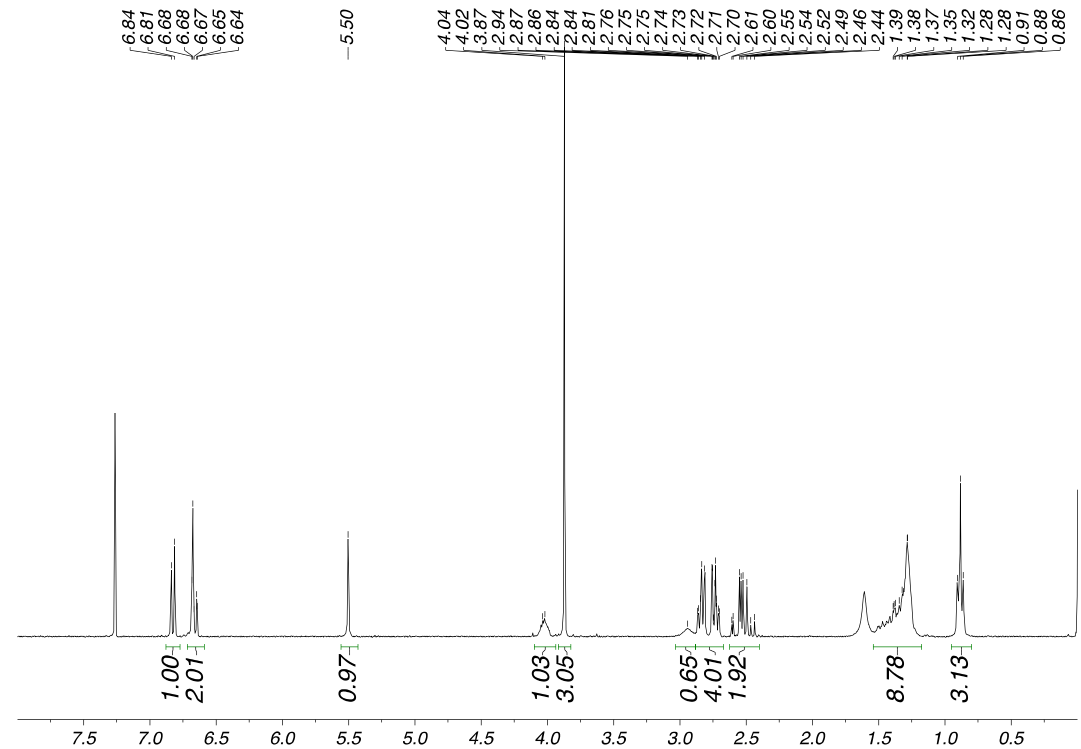

Figure S1. ^{1}H NMR spectrum of gingerols in $CDCl_3$. Data is presented in chemical shift values from tetramethylsilane (TMS); the solvent peak (^{1}H δ 7.26 ppm) was used as internal reference

Data was compared with literature reports for pure 6-gingerol, as shown in table S1.

Table S1. ^{1}H NMR data of 6-gingerol by Z. Gan et al (2016) and herein obtained gingerols

	**6-Gingerol** 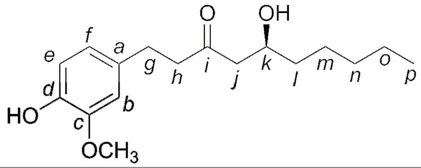		**Gingerols extract**	
	**δ (ppm)**	**Integration**	**δ (ppm)**	**Integration**
H*p*	0.86	3.0341	0.86-0.91	3.13
H*l*, H*m*, H*n*, H*o*	1.25-1.59	8.4582	1.28-1.39	8.78
H*j*	2.44-2.59	2.0309	2.44-2.61	1.92
H*h*	2.70-2.76	1.9902	2.70-2.87	4.01
H*g*	2.81-2.87	1.9823		
H*k*	2.93	0.9519	2.94	0.65
O-CH$_3$	3.87	2.9818	3.87	3.05
k-**OH**	4.05	1.000	4.02-4.04	1.03
d-**OH**	5.50	0.9689	5.50	0.97
H*e*, H*f*	6.64-6.71	1.9703	6.64-6.68	2.01
H*b*	6.78-6.84	0.9798	6.81-6.84	1.00

Note that integrations match the expected values for all protons with the exception of those of H*l*, H*m*, H*n*, H*o* that reveal a higher number of CH$_2$ protons. This denotes the contribution of 8- and 10-gingerols, also present in our sample of gingerols extract.

S2. Mass spectrometry

The TOF MS ES$^+$ spectrum of the gingerols sample is presented in the Figure S2 (next page).

The three main peaks are interpreted assuming the same ionisation mode for all of them. They can be attributed to the monocations formed by 6-, 8-, and 10-gingerols and an adsorbed sodium cation. By subtraction of the sodium mass (Mr (Na^+) = 22), it is known that m/z = 317.2 corresponds to 6-gingerol, m/z = 345.2 corresponds to 8-gingerol and m/z = 373.3 to 10-gingerol. Relative to the percentages obtained in the graph, where 100% corresponds to 6-gingerol and the percentages of 8-gingerol and 10-gingerol are 35% and 47.5% respectively. This means that the extract has 54.05% of 6-gingerol, 19.45% of 8-gingerol and 26.5 % of 10-gingerol, which implicates a molecular mass of 314.71 g/mol, as shown in Table S2.

Table S2. Ginger rhizome extract composition.

**m/z**	**Compound**	**MW (g/mol)**	**Quantity (%)**	
317.2	6-gingerol	294.38	54.05	159.11
345.2	8-gingerol	322.44	19.45	62.71
373.3	10-gingerol	350.49	26.50	92.88
Total Molecular Weight (g/mol)				314.71

Reference

Gan, Z.; Liang, Z.; Chen, X.; Wang, Y.; Li, M.; Ni, Y. Separation and preparation of 6-gingerol from molecular distillation residue of Yunnan ginger rhizomes by high-speed counter-current chromatography and the antioxidant activity of ginger oils *in vitro*. *J. Chromatog. B* **2016**, *1011*, 99–107.

Figure S2. Mass spectrum obtained for gingerols

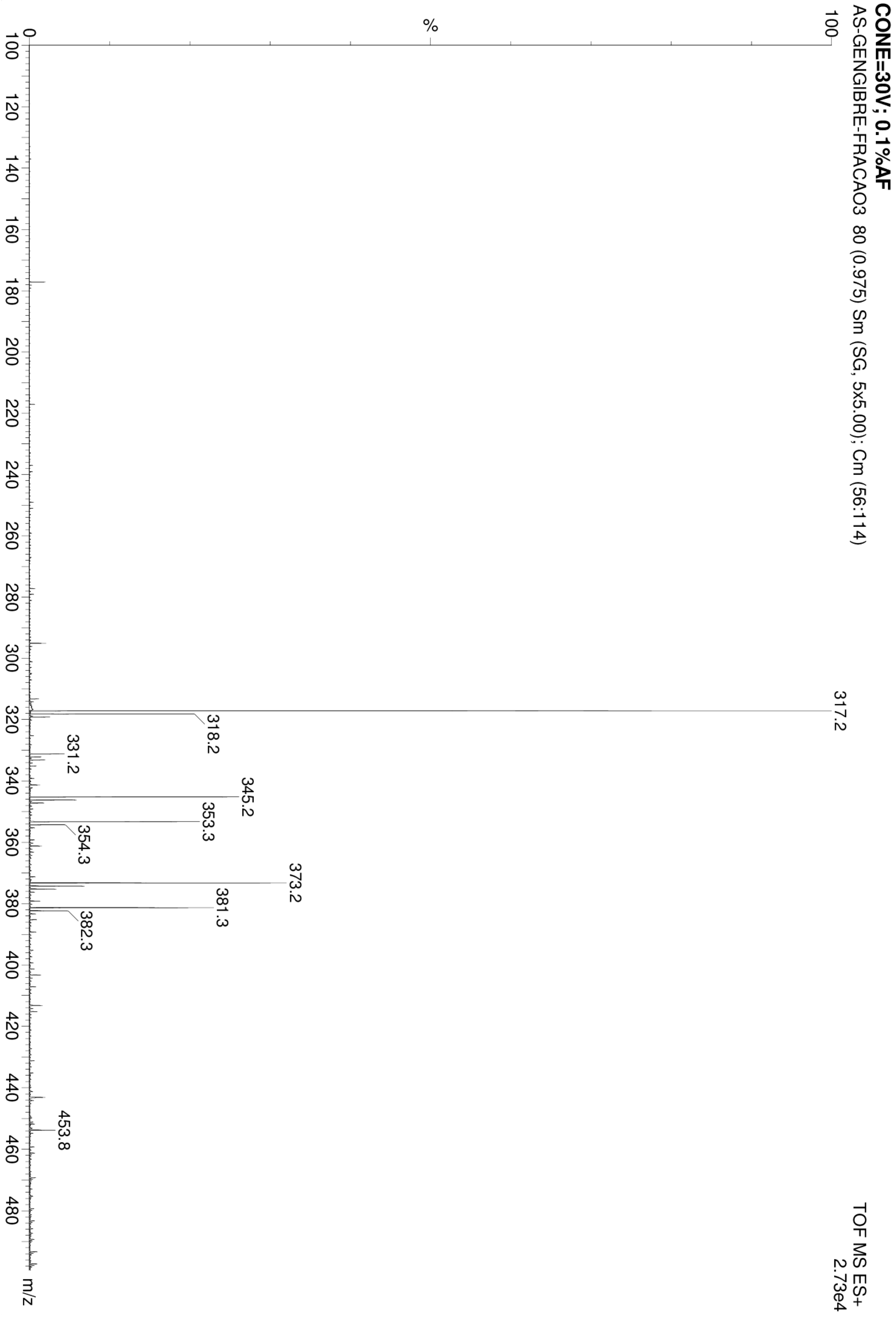

Supplement: Supplementary file 1 [file biomolecules-10-00344-s001.pdf]
